# Neuropsychological impairment in post-COVID condition individuals with and without cognitive complaints

**DOI:** 10.3389/fnagi.2022.1029842

**Published:** 2022-10-20

**Authors:** Mar Ariza, Neus Cano, Bàrbara Segura, Ana Adan, Núria Bargalló, Xavier Caldú, Anna Campabadal, Maria Angeles Jurado, Maria Mataró, Roser Pueyo, Roser Sala-Llonch, Cristian Barrué, Javier Bejar, Claudio Ulises Cortés, NAUTILUS-Project Collaborative Group, Vanesa Arauzo, Carme Junqué, Maite Garolera

**Affiliations:** Consorci Sanitari de Terrassa (CST), Terrassa, Spain. Hospital Sant Joan Despí Moisès Broggi, Consorci Sanitari Integral. Hospital Universitari Arnau de Vilanova, Lleida, Spain. Hospital Universitari de Santa Maria, Lleida, Spain. Consorci Sanitari Alt Penedès-Garraf, Vilafranca de Penedés, Barcelona, Spain. Hospital Verge de la Cinta, Tortosa, Tarragona, Spain. Fundació Sant Hospital de la Seu d’Urgell, La Seu d’Urgell, Lleida, Spain. Consorci Hospitalari de Vic, Vic, Barcelona, Spain. Servei de Malalties Infeccioses, Fundació Lluita contra les Infeccions – Hospital Universitari Germans Trias i Pujol, Badalona, Barcelona, Spain. Hospital Universitari de Bellvitge, L’Hospitalet de Llobregat, Barcelona, Spain. Hospital Universitari Mútua Terrassa, Terrassa, Barcelona, Spain. Badalona Serveis Assistencials, Badalona, Barcelona, Spain. Institut d’Assistència Sanitària, Girona, Spain. Fundació Salut Empordà, Figueres, Girona, Spain. Fundació Hospital de Puigcerdà, Puigcerdà, Girona, Spain. Hospital Universitario Central de la Cruz Roja San José y Santa Adela, Madrid, Spain. Servei Andorrà d’Atenció Sanitària (SAAS), Andorra.; ^1^Medical Psychology Unit, Department of Medicine, University of Barcelona, Barcelona, Spain; ^2^Institute of Neurosciences, University of Barcelona, Barcelona, Spain; ^3^Clinical Research Group for Brain, Cognition and Behavior, Consorci Sanitari de Terrassa (CST), Terrassa, Spain; ^4^Institut d’Investigacions Biomèdiques August Pi i Sunyer (IDIBAPS), Barcelona, Spain; ^5^Centro de Investigación Biomédica en Red sobre Enfermedades Neurodegenerativas (CIBERNED), Barcelona, Spain; ^6^Department of Clinical Psychology and Psychobiology, University of Barcelona, Barcelona, Spain; ^7^Diagnostic Imaging Centre, Hospital Clínic de Barcelona, University of Barcelona, Barcelona, Spain; ^8^Centro de Investigación Biomédica en Red de Salud Mental (CIBERSAM), Instituto de Salud Carlos III, Barcelona, Spain; ^9^Institut de Recerca Sant Joan de Déu (IRSJD), Barcelona, Spain; ^10^Department of Biomedicine, University of Barcelona, Barcelona, Spain; ^11^Centro de Investigación Biomédica en Red en Bioingeniería, Biomateriales y Nanomedicina (CIBER-BBN), Barcelona, Spain; ^12^Department of Computer Science, Universitat Politècnica de Catalunya – BarcelonaTech, Barcelona, Spain; ^13^Neuropsychology Unit, Consorci Sanitari de Terrassa (CST), Terrassa, Spain

**Keywords:** COVID-19, post-COVID-19 condition, NeuroCOVID, neuropsychological test, cognitive function

## Abstract

One of the most prevalent symptoms of post-COVID condition is cognitive impairment, which results in a significant degree of disability and low quality of life. In studies with large sample sizes, attention, memory, and executive function were reported as long-term cognitive symptoms. This study aims to describe cognitive dysfunction in large post-COVID condition individuals, compare objective neuropsychological performance in those post-COVID condition individuals with and without cognitive complaints, and identify short cognitive exams that can differentiate individuals with post-COVID symptoms from controls. To address these aims, the Nautilus project was started in June 2021. During the first year, we collected 428 participants’ data, including 319 post-COVID and 109 healthy controls (18–65 years old) from those who underwent a comprehensive neuropsychological battery for cognitive assessment. Scores on tests assessing global cognition, learning and long-term memory, processing speed, language and executive functions were significantly worse in the post-COVID condition group than in healthy controls. Montreal Cognitive Assessment, digit symbol test, and phonetic verbal fluency were significant in the binomial logistic regression model and could effectively distinguish patients from controls with good overall sensitivity and accuracy. Neuropsychological test results did not differ between those with and without cognitive complaints. Our research suggests that patients with post-COVID conditions experience significant cognitive impairment and that routine tests like the Montreal Cognitive Assessment, digit symbol, and phonetic verbal fluency test might identify cognitive impairment. Thus, the administration of these tests would be helpful for all patients with post-COVID-19 symptoms, regardless of whether cognitive complaints are present or absent.

**Study registration:**

www.ClinicalTrials.gov, identifiers NCT05307549 and NCT05307575.

## Introduction

Since the World Health Organization (WHO) declared COVID-19 a pandemic in March 2020, it has been an ongoing challenge for healthcare systems worldwide. Until the development and implementation of vaccines, most efforts focused on the disease’s acute phase. With a large part of the population now vaccinated and more defined treatment strategies being made available, concerns about mortality have somewhat decreased. However, a significant number of people who have been infected have persistent symptoms, causing disability or decreased quality of life. The post-COVID-19 condition (PCC) occurs approximately 3 months from the onset, with symptoms lasting for at least 2 months, cannot be attributed to alternative diagnoses, and impact everyday functioning (Soriano et al., 2022). PCC is more common in the more severe COVID-19 forms, but it still affects patients who are not hospitalized (Chen et al., 2022). Regarding age, PCC affects both young and old persons, even though it occurs more frequently in the elderly (Daugherty et al., 2021; Cohen et al., 2022). Moreover, women are more likely than men to have PCC (Davis et al., 2021).

PCC is characterized by a wide variety of symptoms, either fixed or fluctuating. They may arise for the first time or continue from the acute phase in a milder or more severe form (Soriano et al., 2022). The most prevalent symptoms include fatigue, pain, headaches, dyspnea, changed smell and taste, cognitive impairment, and mental health issues. These symptoms most likely belong to numerous syndromes, resulting from various pathophysiological processes across the disease spectrum. Proposed mechanisms to explain the pathogenesis of PCC include organ damage in the acute infection phase, a persistent hyperinflammatory state, viral activity associated with a host viral reservoir, or an incompetent antibody response (Proal and VanElzakker, 2021). In addition to acute disease, other factors such as previous comorbidities (Cellai and O’Keefe, 2020), psychological disorders (Mazza et al., 2020), or lifestyle changes due to the pandemic (Galea et al., 2020) may explain this chronicity.

Cognitive dysfunction is one of the most reported symptoms of PCC and generates more significant disability or a decrease in quality of life. In long-COVID studies, brain fog and cognitive dysfunction are self-reported in around 70–80% of patients (Davis et al., 2021; Guo et al., 2022; Ziauddeen et al., 2022). Patients with critical forms of the disease, severe neurological manifestations, or older individuals are more likely to have long-term cognitive dysfunction, according to previous investigations involving patients who experienced acute respiratory distress syndrome from causes other than the SARS-CoV-2 virus (Hopkins et al., 2005; Denke et al., 2018). However, for unknown reasons, cognitive dysfunction also occur frequently in young people with non-severe forms of COVID-19 (Davis et al., 2021).

Initial neuropsychological evaluations supported people’s self-reported data. Attention, memory, and executive function were impaired in participants discharged from the hospital or who recently recovered from a moderate or mild case of COVID-19 (Almeria et al., 2020; Woo et al., 2020; Zhou et al., 2020; Silva et al., 2021). From an online assessment platform, nine computerized cognitive tests were employed in a prospective evaluation with a sample size of more than 84,000 participants. In tests of reasoning, problem-solving, spatial planning, and target detection, 12,689 people who suspected they had COVID-19 performed worse than those who did not report the disease. Depending on the severity of COVID-19, these cognitive deficiencies had varying degrees of impact on several tests (Hampshire et al., 2021).

Studies that focused on long-term cognitive symptoms have confirmed the initial findings with case studies or small samples. A study on 740 people conducted 7 months after the COVID-19 diagnosis using cut-off scores [defined as a *Z*-score ≤ 1.5 standard deviation (SD) below measure-specific age-, educational level-, and sex-adjusted norm of classical standardized tests] found impairments in all domains assessed, ranging from 10% in attention and working memory to 24% in verbal encoding (Becker et al., 2021). Another study on 66 PCC subjects selected according to cognitive complaints also found low scores across domains ranging from 15 to 52% in attention and 12 to 32% in executive functions (García-Sánchez et al., 2022). However, both these studies lacked a control group. Delgado-Alonso et al. (2022) compared the results of a paper and pencil and computerized testing of a sample of 50 people with post-COVID cognitive complaints with 50 healthy controls (HCs). They found impaired attention-concentration, episodic memory, visuospatial processing, and executive functions (Delgado-Alonso et al., 2022). Guo et al. (2022) compared 181 people with PCC and 185 HCs by using several online experimental tasks, and only found impairments in memory but not executive functions or language.

Despite existing research, more data is needed to comprehend COVID-19’s impacts on cognition. This study aims first to describe the cognitive dysfunctions in a large PCC and compare them with a HC group. Our second aim is comparing the objective performance in individuals with and without subjective cognitive complaints. We expect to find more affectation in PCC individual with cognitive complaints. Finally, we aim to detect the neuropsychological tests that better discriminate patients from controls, to be proposed as short cognitive screenings. We selected a neuropsychological battery using instruments typically utilized in clinical settings, but we also included the recognition of emotions because of its sensitivity to the orbital cortex (Adolphs, 2002). To date, no study has been published that evaluates social cognition in PCC individuals. We expect to find more affectations in emotion recognition in PCC group.

## Materials and methods

### Participants

The sample comprised 428 participants from the Nautilus Project (ClinicalTrials.gov IDs: NCT05307549 and NCT05307575). Three hundred and nineteen participants with PCC and 109 HCs were evaluated at the Neuropsychology and COVID-19 Units across 16 hospitals in Catalonia, Madrid, and Andorra, coordinated by the Consorci Sanitari de Terrassa (Terrassa, Barcelona, Spain). The inclusion criteria for the PCC group were as follows: (a) confirmed diagnosis of COVID-19 according to WHO criteria with signs and symptoms of the disease during the acute phase; (b) at least 12 weeks after infection; and (c) age between 18 and 65 years. The exclusion criteria were: (a) established diagnosis before COVID-19 disease of psychiatric, neurological, neurodevelopmental disorder, or systemic pathologies known to cause cognitive deficits, and (b) motor or sensory alterations that impede the neuropsychological examination. The HCs did not have COVID-19 (no positive test or compatible symptoms), and the same exclusion criteria were applicable to the PCC group. All participants were native Spanish speakers.

### Procedure

The overall procedure consisted of two sessions. In the first session, various questionnaires were administered to collect information about demographic factors, previous comorbidities, and data on COVID-19. Participants provided information on their age, sex, formal education, citizenship, ethnicity, profession, and income. They were questioned about their medical history and behavior related to their health. Moreover, they were also asked about their COVID-19 experience, including their symptoms, treatment, hospitalization, and time since diagnosis. We also collected information on their post-COVID symptoms, including cognitive ones.

Each participant underwent a cognitive assessment with a comprehensive neuropsychological battery in the second session. We used the Montreal Cognitive Assessment (MoCA) as a general cognitive screening tool (Nasreddine et al., 2005; Ojeda et al., 2016). The Matrix subtest from the Wechsler Adult Intelligent Scale (WAIS) III was used to assess abstract reasoning (Wechsler, 1999). To assess verbal memory, we used the Spanish version of Rey’s Auditory Verbal Learning Test (RAVLT) (Schmidt, 1996; Alviarez-Schulze et al., 2022). Visual memory was evaluated with the 30-min delayed recall test from the Rey–Osterrieth Complex Figure Test (ROCF) (Meyers and Meyers, 1996). The copy trial of the ROCF evaluated the visuo-constructive abilities. The WAIS-III Digit Span subtest was used to measure verbal attention (digit span forward) and working memory (digit span backward) (Wechsler, 1999). Visual scanning, tracking, and motor speed were assessed by the digit symbol test from the WAIS-III (Wechsler, 1999). Parts A and B of the Trail Making Test (TMT) were administered to measure visual scanning, motor speed and attention, and mental flexibility (Reitan, 1958). The Controlled Oral Word Association Test (COWAT) (Benton and Hamsher, 1989; Peña-Casanova et al., 2009) was used to evaluate verbal fluency and language. The number of words beginning with the letters P, M, and R recalled in 1 min was recorded. Semantic fluency was evaluated using the category “animals” (Ardila et al., 2006). The number of correct animals recalled in 1 min was considered. The Stroop test consists of three subtests: words, colors, and color words that conflict with the color in which they are presented. Here, the interference score was calculated as a measure of cognitive inhibitory control (Golden, 2005). The Boston Naming Test (BNT) was used to evaluate language (Allegri et al., 1997). Emotion recognition was assessed with the Reading the Mind in the Eye Test (Fernández-Abascal et al., 2013). The Word Accentuation Test (TAP) was included as an estimate of premorbid IQ (Gomar et al., 2011). In addition to cognitive measures, we used the Chalder Fatigue Scale (CFQ) (Jackson, 2014) to assess fatigue, the Generalized Anxiety Disorder 7-item scale (GAD-7) (Spitzer et al., 2006; García-Campayo et al., 2010) to assess anxiety, and the Patient Health Questionnaire-9 (PHQ-9) (Diez-Quevedo et al., 2001; Kroenke et al., 2001) to assess depression. All evaluations were performed by trained neuropsychologists.

The recruitment was carried out between June 2021 and June 2022. The study was conducted with the approval of the Drug Research Ethics Committee (CEIm) of Consorci Sanitari de Terrassa (CEIm code: 02-20-107-070) and the Ethics Committee of the University of Barcelona (IRB00003099). All participants provided written informed consent.

### Statistical analyses

Descriptive statistics were conducted for all the variables of the study. Group differences in demographics were examined by conducting two-tailed Student’s *t*-tests. The Fisher’s exact test assessed a comparison of binarized measures between the two groups. One-way analysis of covariance (ANCOVA) was performed to determine differences in cognitive functioning among groups, including age, sex, education, and estimated IQ as nuisance variables. Graphical representations and descriptive statistics were used to study the assumptions. The effect size was calculated using the value partial eta squared ($\eta_{p}^{2}$). We used logistic regression to assess the additive contribution of neuropsychological variables in classifying the PCC and HC. We used age, years of education, and sex as covariables. Results were presented as odds ratios with 95% confidence intervals (CIs). We reported the accuracy, sensitivity, and specificity, and positive and negative predictive values. The area under the ROC curve (AUROC) was also calculated. Analyses were performed using IBM SPSS Statistics 27.0 (IBM Corporation, Armonk, NY, USA) and R Statistical Software (version 4.2.0; The R Foundation for Statistical Computing Platform). The critical level for statistical significance was set at α = 0.05. A Bonferroni adjustment was made for ANCOVA analyses such that statistical significance was accepted when *p* < 0.0025.

## Results

Table 1 shows the socio-demographic characteristics and comorbidities of the PCC and HC groups. The PCC group had a higher proportion of women (77 vs. 62%), were older, had less formal education, and had a lower estimated IQ than the control group. Therefore, age, sex, educational level, and estimated IQ were covariates in comparing cognitive results between the two groups. Compared to the HC group, respiratory disease, high blood pressure, and obesity were more prevalent among PCC participants. On average, patients had a positive test 320 days before their neuropsychological evaluation (SD = 156.66 days, range: 84–795 days).

**TABLE 1:** Socio-demographic characteristics and comorbidities for the PCC and HC groups.

	PCC	HC		
	*n* = 319 *M* (SD) Range	*n* = 109 *M* (SD) Range	*t*	*p*
Age (years)	49.06 (9.13) 24–65	46.10 (9.31) 23–62	2.901	0.004
Education (years)	13.78 (3.34) 8–20	15.57 (2.93) 8–20	5.300	<0.001
IQ estimation*	101.51 (7.87) 85–116	104.79 (6.58) 85–116	4.235	<0.001
	
	***n* (%)**	***n* (%)**	**χ ^2^**	***P***
	
Sex (% female)	84 (77.7%)	68 (62.4%)	7.817	0.005
Change of employment status (post-COVID)	126 (39.5%)	9 (8.3%)	36.722	<0.001
***Previous comorbidities***				
Heart disease	11 (3.5%)	3 (2.8%)		
Respiratory disease	40 (12.5%)	5 (4.6%)	6.635	0.036
Chronic kidney disease	3 (0.9%)	0		
High blood pressure	47 (14.7%)	5 (4.6%)	9.055	0.011
Dyslipidemia	46 (14.4%)	11 (10.1 %)	2.430	0.297
Diabetes mellitus	13 (4.1%)	3 (2.8%)		
Obesity	99 (31.3%)	16 (14.7%)	12.469	0.002
Chronic liver disease	10 (3.2%)	0		
Tobacco smoking	22 (7.0%)	27 (24.8%)	26.348	<0.001

PCC, post-COVID condition; HC, healthy control; M, mean; SD, standard deviation.

*By means of Word Accentuation Test.

Regarding the severity of the disease, 150 (47%) PCC patients were hospitalized, of which 77 (51.3%) were admitted to the intensive care unit (ICU). The remaining 169 (53%) individuals with PCC were outpatients and had a mild illness at home. Of those, 139 (82.2%) had disturbance of activities of daily living, and 30 (17.8%) continued to carry out their activities as usual. Table 2 shows symptoms reported by participants with PCC at the time of assessment. Fatigue, pain, and headache were the most reported post-COVID general symptoms, whereas cognitive complaints, depressive, and anxiety manifestations were the most frequently reported among the neuropsychiatric symptoms.

**TABLE 2:** Post-COVID-19 condition reported symptoms at the time for neuropsychological assessment (*N* = 319).

Symptom	Cases (%)
Fatigue	209 (65.5)
Joint pain/body aches	140 (43.9)
Headaches	136 (43.3)
Dyspnea on exertion	122 (38.2)
Limb weakness	98 (30.7)
Paresthesia	87 (27.3)
Altered smell	98 (30.7.1)
Chest pain	70 (21.9)
Altered taste	64 (20.1)
Dizziness	68 (21.3)
Cough	53 (6.6)
Menstrual cycle alteration	8 (10.3)*
Sore throat	37 (11.6)
Nasal congestion	36 (11.3)
Loss of appetite	33 (10.3)
Dermatologic issues	27 (8.5)
Conjunctival congestion	24 (7.5)
Diarrhea	23 (7.2)
Loss of hair	22 (6.9)
Nausea	18 (5.6)
***Neuropsychiatric symptoms***	
Overall cognitive complains (subjective)	123 (38.6)
Memory deficits	110 (34.5)
Lack of concentration	106 (33.2)
Brain fog	97 (30.4)
Problems with language	79 (24.7)
Problems with executive functioning	73 (22.9)
Depressive symptoms	101 (31.7)
Anxiety	98 (30.7)
Post-traumatic stress	43 (13.5)
Difficulty sleeping	40 (12.5)
Obsessive-compulsive symptoms	16 (5)
Psychotic symptoms	3 (0.94)

PCC, post-COVID condition.

*% women < 45 years (*n* = 78).

**FIGURE 1:**
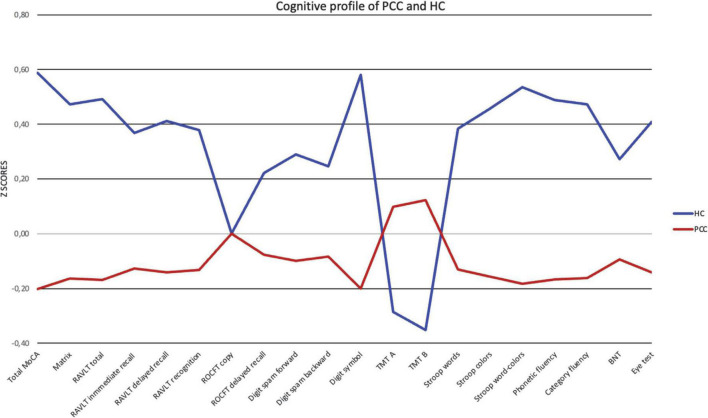
Cognitive profile for PCC and HC. Healthy controls (HC) in blue, PCC in red. Data are presented as *Z*-scores. Lower *Z*-scores indicate poorer performance, except for TMT (time), where lower *Z*-scores mean better performance.

**FIGURE 2:**
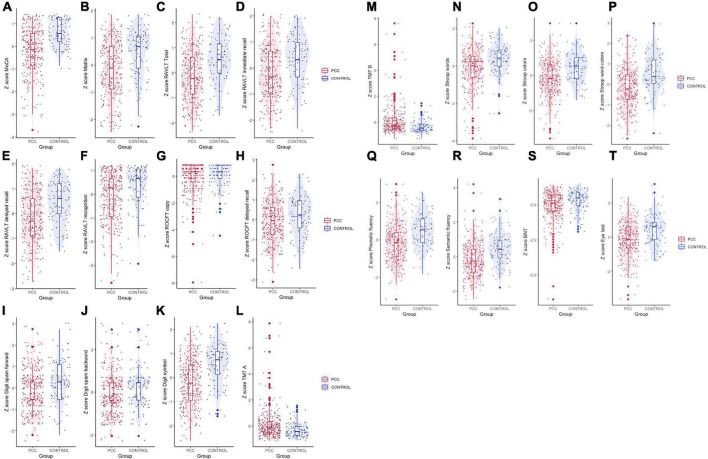
Violin plot for cognitive variables of PCC and HC groups. Data are presented as *Z*-scores. **(A)** MoCA, **(B)** matrix reasoning, **(C)** Rey’s Auditory Verbal Learning test (RAVLT) total (sum of 5 trials), **(D)** RAVLT immediate recall, **(E)** RAVLT delayed recall, **(F)** RAVLT recognition, **(G)** Rey–Osterrieth Complex Figure Test (ROCFT) copy, **(H)** ROCFT delayed recall, **(I)** digit spam forward, **(J)** digit spam backward, **(K)** digit symbol test (coding), **(L)** Trail Making Test (TMT) A, **(M)** TMT B; **(N)** Stroop test words, **(O)** Stroop test colors, **(P)** Stroop test word-colors (interference), **(Q)** phonetic fluency (PMR), **(R)** semantic fluency (animals), **(S)** Boston Naming Test (BNT), and **(T)** Reading the Mind in the Eyes test (Eye test).

After adjusting for covariates and considering the Bonferroni correction for the number of comparisons (which leaves us with a significance level of *p* = 0.0025), there was a statistically significant poor performance of PPC group in MoCA, matrix reasoning, RAVLT sum, RAVLT delayed recall, digit symbol, Stroop words, Stroop colors, Stroop interference, phonetic fluency, and semantic fluency than in HC group (Table 3 and Figures 1, 2).

**TABLE 3:** Adjusted* means for the neuropsychological variables for PCC and HC groups.

	PCC		HC				
	*N*	*M*_adj_ (SE)	*N*	*M*_adj_ (SE)	*F*	*p*	$\eta_{p}^{2}$
MoCA	**310**	**26.02 (0.14)**	**106**	**27.54 (0.24)**	**28.196**	**<0.001**	**0.064**
Matrix reasoning	**308**	**16.09 (0.25)**	**107**	**17.97 (0.43)**	**13.715**	**<0.001**	**0.032**
RAVLT sum	**311**	**44.24 (0.47)**	**107**	**48.09 (0.81)**	**16.703**	**<0.001**	**0.039**
RAVLT immediate recall	311	8.88 (0.15)	107	9.58 (0.26)	5.436	0.020	0.013
RAVLT delayed recall	**310**	**8.77 (0.17)**	**107**	**9.83 (0.29)**	**9.982**	**0.002**	**0.023**
RAVLT recognition	308	12.16 (0.13)	107	12.91 (0.23)	7.696	0.006	0.018
ROCFT copy	311	32.91 (0.21)	107	32.83 (0.35)	0.050	0.815	0.000
ROCFT delayed recall	311	18.93 (0.32)	107	19.43 (0.56)	0.532	0.466	0.001
Digit span forward	311	5.58 (0.06)	106	5.94 (0.11)	7.424	0.007	0.018
Digit span backward	313	4.42 (0.06)	106	4.66 (0.11)	3.346	0.068	0.008
Digit symbol	**310**	**64.39 (0.93)**	**107**	**73.82 (1.62)**	**24.743**	**<0.001**	**0.058**
TMT-A	310	38.053 (1.1)	107	32.92 (2.02)	5.032	0.025	0.012
TMT-B	306	88.39 (3.13)	107	71.64 (5.40)	7.180	0.008	0.017
Stroop words	**309**	**93.16 (1.18)**	**106**	**100.72 (2.06)**	**10.166**	**0.002**	**0.024**
Stroop colors	**309**	**64.08 (0.75)**	**106**	**70.42 (1.32)**	**17.293**	**<0.001**	**0.040**
Stroop word-colors	**309**	**38.38 (0.57)**	**106**	**48.85 (0.99)**	**23.065**	**<0.001**	**0.053**
Phonetic fluency (PMR)	**312**	**41.66 (0.65)**	**107**	**47.08 (1.13)**	**17.122**	**<0.001**	**0.039**
Semantic fluency (animals)	**311**	**20.94 (0.29)**	**107**	**23.28 (0.50)**	**15.818**	**<0.001**	**0.037**
BNT	311	52.09 (0.26)	107	52.89 (0.46)	2.055	0.152	0.005
Eye test	310	22.26 (0.20)	107	23.47 (0.35)	8.509	0.004	0.020

PCC, post-COVID condition; HC, healthy control; MoCA, Montreal Cognitive Assessment; RAVLT, Rey’s Auditory Verbal Learning Test; ROCFT, Rey–Osterrieth Complex Figure Test; TMT, Trail Making Test; BNT, Boston Naming Test.

*Adjusted by years of education, estimated IQ, age, and sex.

$\eta_{p}^{2}$ effect size is as follows: $\eta_{p}^{2}=0.009$, small; $\eta_{p}^{2}=0.059$, medium; $\eta_{p}^{2}=0.139$, large.

The results after Bonferroni correction are indicated in bold font (*p* < 0.0025).

The PCC group showed statistically significant higher scores of CFQ (PCC: mean = 6.21, SD = 4.33 vs. HC: mean = 1.73, SD = 3.07; *t* = −9.730, *p* < 0.001, *d* = 1.104), GAD-7 (PCC: mean = 6.73, SD = 5.55 vs. HC: mean = 3.18, SD = 3.12; *t* = −6.178, *p* < 0.001, *d* = 0.702), and PHQ-9 (PCC: mean = 9.13, SD = 6.64 vs. HC: mean = 3.08, SD = 2.79; *t* = 9.004, *p* < 0.001, *d* = 1.023) than those of the HC group. We reanalyzed the data by taking fatigue, anxiety, and depression scale scores as covariates. After adjusting for these variables, there was a statistically significant poor performance of the PPC group in MoCA (*F* = 10.120; *p* = 0.002; partial η^2^ = 0.025), RAVLT sum (*F* = 4.843; *p* = 0.028; partial η^2^ = 0.012), digit symbol (*F* = 7.448; *p* = 0.007; partial η^2^ = 0.019), Stroop word-colors (*F* = 5.757; *p* = 0.017; partial η^2^ = 0.015), phonetic fluency (*F* = 5.802; *p* = 0.016; partial η^2^ = 0.015), semantic fluency (*F* = 6.055; *p* = 0.014; partial η^2^ = 0.015), and Reading the Mind in the Eyes test (*F* = 7.576; *p* = 0.006; partial η^2^ = 0.019). However, no result remained statistically significant after Bonferroni correction (see Supplementary Table).

**FIGURE 3:**
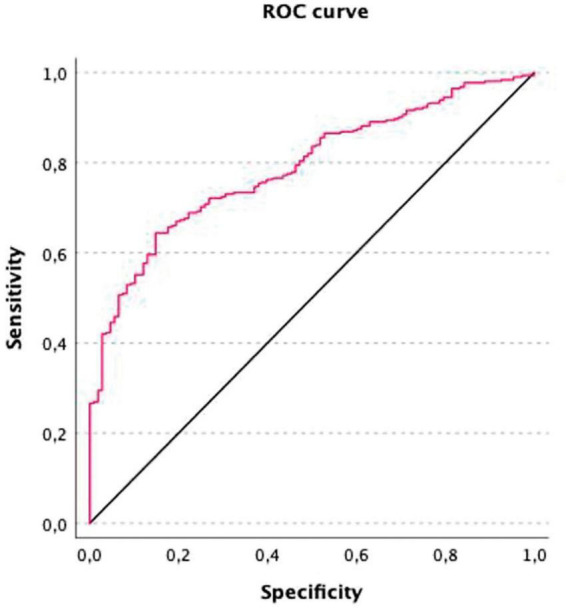
Receiver operating characteristic (ROC) curve for discrimination between PCC and controls based on MoCA, digit symbol, and phonetic fluency. The model’s total diagnostic accuracy is summarized by the AUROC. A value of 0 represents a completely inaccurate test, and a value of 1 represents a completely accurate test. AUC = 0.788 (95% CI: 0.744–0.832).

We focused on the neuropsychological variables that better distinguished patients and controls. We performed binomial logistic regression using the group as the outcome and the significant variables after the Bonferroni correction in the comparison between the two groups as predictors. We added demographic variables (age, years of formal education, and sex) as covariables. Linearity of the continuous variables for the logit of the dependent variable was assessed using the Box–Tidwell procedure (Box and Tidwell, 1962). A Bonferroni correction was applied using all 19 terms in the model, resulting in statistical significance being accepted when *p* < 0.00263 (Tabachnick and Fidell, 2014). Based on this assessment, all continuous independent variables were found to be linearly related to the logit of the dependent variable. The logistic regression model was statistically significant (χ^2^_(3)_ = 87.862, *p* < 0.001). The link test was nonsignificant, indicating good model specification. The Hosmer–Lemeshow goodness-of-fit test was non-significant, indicating good model fit (χ^2^_(8)_ = 12.639, *p* = 0.125). The model explained 28.0% (Nagelkerke *R*^2^) of the variance. Of the nine predictor variables, three made significant contributions to the model: total MoCA [odds ratio (OR) = 0.731], digit symbol test (OR = 0.973), and phonetic fluency (OR = 0.977) (Table 4). The model demonstrated overall classification accuracy of 74.5%, with a sensitivity of 89.9% and a specificity of 30.6%. The positive predictive value was 78.63%, and the negative predictive value was 51.56%. The AUROC 0.788 (95% CI: 0.744–0.832), which is an acceptable level of discrimination (Hosmer et al., 2013; Figure 3).

**TABLE 4:** Logistic regression classifying participants in PCC and HC groups based on significant neuropsychological results.

	*B*	SE	Wald	df	*p*	Odds ratio	95% CI for odds ratio	
							Lower	Upper
Total MoCA	−0.313	0.065	22.927	1	<0.001	0.731	0.643	0.831
Digit symbol	−0.027	0.008	11.382	1	<0.001	0.973	0.958	0.989
Phonetic fluency	−0.023	0.011	4.782	1	0.029	0.977	0.956	0.998
Constant	12.529	1.792	48.904	1	<0.001	276,250.358		

MoCA, Montreal Cognitive Assessment.

To evaluate whether the cognitive complaint is a determining factor in worse neuropsychological performance, we formed two groups: subjects who reported cognitive complaints (CC) (*n* = 123, 38.6%) and those who did not notice cognitive changes (NCC) (*n* = 196). The groups were similar in age (NCC: mean = 49.11, SD = 9.829 vs. CC: mean = 48.97, SD = 7.941); education (NCC: mean = 13.68, SD = 3.266 vs. CC: mean = 13.93, SD = 3.461), and estimated IQ (NCC: mean = 101.86, SD = 8.162 vs. CC: mean = 100.95, SD = 7.382), but the CC group had significantly more days since the positive test than the NCC group (CC: mean = 370, SD = 199.329, NCC: mean = 288, SD = 111.748; *t* = −4.193, *p* < 0.001, *d* = 0.546). Additionally, the CC group had 87 (70.7%) women compared to the 112 (57%) in the NCC group (χ^2^_(1)_ = 5.947, *p* = 0.015). There were no differences in GAD-7 scores (NCC: mean = 6.44, SD = 5.67 vs. CC: mean = 7.20, SD = 5.35) between groups. However, the scores of the CFQ (NCC: mean = 7.94, SD = 6.39 vs. CC: mean = 11.06, SD = 6.62) and the PHQ-9 (NCC: mean = 5.37, SD = 4.37 vs. CC: mean = 7.60, SD = 9.91) were significantly higher in the CC group than in the NCC group (CFQ: *t* = −4.488, *p* < 0.001, *d* = 0.530; PHQ-9: *t* = −4.065, *p* < 0.001, *d* = 0.481). Thus, we compared the neuropsychological performance of both groups controlling for sex, days of evolution, fatigue, and depression. We did not find significant differences at the Bonferroni level in the neuropsychological variables between participants with cognitive complaints and those without.

## Discussion

The present study aimed to characterize the cognitive impairment of a large sample of participants with PCC. Previous studies have shown that people who had COVID-19 performed worse than comparable healthy subjects in all cognitive domains, namely attention, executive functions, memory, and language (Becker et al., 2021; Delgado-Alonso et al., 2022; García-Sánchez et al., 2022; Guo et al., 2022; Zhao et al., 2022). Compared to the HCs, we found lower functioning of the PCC sample in tests of all domains other than attention and visuoconstructive functions.

Contrary to other authors (Becker et al., 2021; Delgado-Alonso et al., 2022; García-Sánchez et al., 2022), we did not find differences in attention between groups. Performance in TMT-A, a test in the attention domain, was not significant, although it was before the Bonferroni correction. García-Sánchez et al. (2022) highlighted the attentional deficits linked to COVID-19, but they used the CPT, a specific attention test that allow to separate between attentional accuracy and responsiveness speed, to detect a slight decrease in attentional abilities. Processing speed is a key component of attention as most attention tests are speed sensitive. Delgado-Alonso et al. (2022) found impaired attention. However, they collapsed several tests, such as Stroop, Symbol Digits Modalities Test, and reaction time tests, in addition to TMT-A and digit span forward, in the domain named attention and processing speed (Delgado-Alonso et al., 2022). Processing speed was affected also in our PCC group. Similar to us, Becker et al. (2021) measured attention with routine tests in clinical settings. They reported a 10% affectation when taking one standard deviation of the *Z*-score in reference to the HCs. However, this impairment was more prevalent in hospitalized patients, and therefore probably in more severe cases. A total of 24% of our PCC participants underwent critical care, which is risk factor for impairment in attention and processing speed (Hopkins et al., 1999). Neuroinflammatory reactions occur with severe systemic infection, as well as mild COVID-19 infections. A pattern of activated white matter microglia similar to that associated with the chemo-brain has been identified in individuals with SARS-CoV-2 infection (Fernández-Castañeda et al., 2022). PPC patients’ mental processing speed likely stems from impairments in complex brain networks rather than specific dysfunctions. The evidence points to an attentional deficit in PCC patients, but the poor results in several tests potentially reflect processing speed issues.

Regarding the memory domain, we found an obvious impairment of verbal learning similar to other authors (Becker et al., 2021; Delgado-Alonso et al., 2022), but we did not find impaired visuoconstructive functions and visual memory. Chronic inflammation has been linked to neuronal impairment, especially in the hippocampus (Belarbi and Rosi, 2013). It has been suggested that patients with PCC could suffer from a chronic inflammatory condition (Maamar et al., 2022). This could explain these memory problems, especially in those who have had milder forms of COVID-19. In addition, affectations in the hippocampus have been related to memory loss at 3 months post-COVID (Lu et al., 2020).

We found that the performance of the Reading the Mind in the Eyes test also differed between PCCs and controls. However, Bonferroni’s corrections were applied, and the differences did not reach the criteria for significance. To date, no studies have been published that evaluate social cognition in PCC individuals even though impaired social cognition can result in difficulties with social communication (Henry et al., 2006). Surprisingly, the Eye test did not correlate with depression and anxiety scores in our PCC participants. Social cognition is affected in depressed individuals (Nejati et al., 2012; Weightman et al., 2014). It has been proposed that the association between decreased social cognition and psychosocial issues in depressed individuals may be mediated by executive functions (Knight and Baune, 2019). The affectation of emotion recognition found in our sample could be explained by the reduction in gray matter in the orbito-frontal cortex seen in a large-sample of the COVID-19 re-imaging study (Douaud et al., 2022).

The neuropsychological profile observed in our data, which is consistent with the mild executive dysfunction syndrome reported by Bertuccelli et al. (2022) in a recent meta-analysis, indicates that individuals infected with COVID-19 are likely to develop neurodegeneration and dementia in the future. Periodical neuropsychological follow-up of PCC individuals is recommended to control the progression of cognitive deficits. We are unsure whether they will continue, resolve, or worsen. This monitoring will also enable us to ensure that the tests used to identify these deficiencies are the best ones available. In any case, the focus of clinical and research professionals should always be on creating interventions for cognitive stimulation.

Interestingly, our results are significant after removing the effect of many variables and performing the Bonferroni correction for multiple comparisons. Group differences were small-to-medium, as indicated by effect size calculations. Low effect size has also been reported by other authors (Delgado-Alonso et al., 2022; García-Sánchez et al., 2022). However, our results might have clinical relevance despite the small or medium effect size. It is a relatively young sample (<65 years of age) with cognitive impairments, which may affect the functionality. In our sample, we do not use objective measures to evaluate the functionality. However, 39.5% of PCC subjects had employment status changes, compared to 8.3% of HCs. Further investigation that additionally examines the mental health, quality of life, and functionality of PCC patients is needed.

Several studies have revealed that subjects with PCC present high levels of fatigue, depression, and anxiety (Fernández-de-Las-Peñas et al., 2021; Mattioli et al., 2021), which are correlated with cognitive deficits (Mattioli et al., 2021; Delgado-Alonso et al., 2022; García-Sánchez et al., 2022; Whiteside et al., 2022). Our results are consistent with those of previous reports. Fatigue, depression, and anxiety explain part of our sample’s variance in cognitive performance, as evidenced by the reduction of cognitive differences between the PCC and HC groups after controlling for these factors. In PCC patients, depression, anxiety, and executive dysfunction have been found to predict fatigue (Calabria et al., 2022). However, it is unknown how depression and cognitive impairment are related causally. Depression plays a role in poor cognitive function. However, it cannot be ruled out that post-COVID symptoms such as cognitive deficits may cause depression. It is also possible that the same illness process causes cognitive impairment and depression, but more research is required to draw exact conclusions about the connection between depression and cognitive deficits.

We found that the neuropsychological tests that best discriminate between PCC and HCs are the MoCA, digit symbol test, and phonetic fluency. The model obtained differentiates acceptably well, has good sensitivity, and correctly identifies PCCs. Two of the tests showing discrimination power are usual screening tools for mild cognitive impairment (MCI) (Nasreddine et al., 2005; González-Blanch et al., 2011). According to a recent meta-analysis, MoCA has already demonstrated its efficacy in detecting cognitive impairments associated with COVID-19 in the first 6 months (Crivelli et al., 2022). Our results reveal that the sensitivity of MoCA to detect cognitive impairment extends well beyond the first 6 months. Digit symbols are a susceptible test for brain damage. This task has not been related to brain structure or function, rather its deficient performance has been linked to various biological or functional pathologies (Lezak et al., 2012). On the other hand, verbal fluency, both phonetic and semantic, has also been shown to discriminate between people with MCI and healthy people, particularly semantic fluency (McDonnell et al., 2020). Semantic fluency does not appear in our model, but phonetic fluency does. It seems that performance in phonetic fluency tests is more sensitive in discriminating between people with PCC and healthy people. Distinct brain structures are involved in these language processing components: word retrieval in semantic fluency depends on semantic associations and each association’s meaning, whereas phonetic fluency involves uncommonly used procedures requiring more effort (Bayles et al., 1989).

Contrary to what we expected, we found no differences in the neuropsychological performance between participants who presented a cognitive complaint and those who did not. According to Calabria et al. (2022), our scores on depression and fatigue were higher in the cognitive-complaint subjects than in those without it. However, patients with cognitive complaints were not cognitively poorer than patients without them, and their increased complaining may have been due to their high levels of depression and fatigue. Our data suggest that anyone with PCC may have cognitive impairment influencing their functionality and quality of life, even if they do not complain. In fact, Zhao et al. (2022) found poor performance on sustained attention tasks up to 9 months after infection in a sample of people who did not seek post-COVID care. Cognitive function screening should be protocolized in the evaluation of people with PPC, even without cognitive complaints.

When interpreting the results, it is essential to consider the limitations and strengths of the current study. Our control group is not optimal, because we had to control some variables statistically. We aimed to match the PCC sample by age, sex, and education. Enrolling people who have not had the disease proved increasingly difficult. Although we could have used old samples from other studies, we wanted to control for the “pandemic” effect (i.e., lockdowns and stress) so that the control group experienced the same environmental circumstances, with the only difference being that they did not experience the infection. Another limitation refers to the choice of instrument to assess visuoconstructive skills and verbal memory. We used the ROCF test, which was normal for both the copying and memory parts. However, tests used by other authors are better suited to measure visual memory and it is possible that our test has not been adequate enough to assess visual memory impairment in COVID-19 patients. We did not investigate associations between cognitive status and biomarkers of clinical severity (i.e., ferritin or CRP). To understand the pathogenesis of cognitive dysfunction in COVID-19 patients, future studies with bigger samples are required to assess these characteristics.

However, our sample size is reasonably large, representing the full spectrum of severity of COVID-19. Moreover, the sample includes both individuals with and without cognitive complaints. This allows the results to be extrapolated to the entire PCC population. In addition, the selection of the sample has been made by ruling out comorbidities that could cause cognitive impairment, which means that we have a clean sample.

## Conclusion

To conclude, despite the methodological limitations, the results of our study, with a large, representative sample of individuals with PCC and a large HC group, show that people with PCC present significant impairments in global cognition, learning and long-term memory, processing speed, language, and executive functions. Even though it has been almost a year since the COVID positive test, these impairments are still observed. We also provide evidence that cognitive deficits can affect anyone with PCC, regardless of whether they experience cognitive complaints. Further, we believe that all patients with post-COVID-19 symptoms would benefit from the routine use of three assessing tools such as MoCA, digit symbol, and verbal fluency test to rule out cognitive impairment. These tests are currently utilized in research and clinical settings. They are simple to conduct and accurate, making them popular among healthcare professionals and patients alike. Healthcare professionals will find our results to be clinically helpful when evaluating cognition in PCC.

## Data availability statement

The raw data supporting the conclusions of this article will be made available by the authors, without undue reservation.

## Ethics statement

The studies involving human participants were reviewed and approved by the Drug Research Ethics Committee (CEIm) of Consorci Sanitari de Terrassa, Terrassa, Barcelona, Spain (CEIm code: 02-20-107-070) Ethics Committee of the University of Barcelona (IRB00003099). The patients/participants provided their written informed consent to participate in this study.

## Members of the NAUTILUS-Project Collaborative Group

Vanesa Arauzo and Jose A. Bernia, Consorci Sanitari de Terrassa (CST), Terrassa, Spain.

Marta Balague-Marmaña and Berta Valles-Pauls, Hospital Sant Joan Despí Moisès Broggi, Consorci Sanitari Integral.

Jesús Caballero, Hospital Universitari Arnau de Vilanova, Lleida, Spain.

Anna Carnes-Vendrell and Gerard Piñol-Ripoll, Hospital Universitari de Santa Maria, Lleida, Spain.

Ester Gonzalez-Aguado and Carme Tayó-Juli, Consorci Sanitari Alt Penedès-Garraf, Vilafranca de Penedés, Barcelona, Spain.

Eva Forcadell-Ferreres and Silvia Reverte-Vilarroya, Hospital Verge de la Cinta, Tortosa, Tarragona, Spain.

Susanna Forné, Fundació Sant Hospital de la Seu d’Urgell, La Seu d’Urgell, Lleida, Spain.

Anna Bartes-Plan and Jordina Muñoz-Padros, Consorci Hospitalari de Vic, Vic, Barcelona, Spain.

Jose A. Muñoz-Moreno and Anna Prats-Paris, Servei de Malalties Infeccioses, Fundació Lluita contra les Infeccions – Hospital Universitari Germans Trias i Pujol, Badalona, Barcelona, Spain.

Inmaculada Rico and Nuria Sabé, Hospital Universitari de Bellvitge, L’Hospitalet de Llobregat, Barcelona, Spain.

Marta Almeria and Laura Casas, Hospital Universitari Mútua Terrassa, Terrassa, Barcelona, Spain.

Maria José Ciudad and Anna Ferré, Badalona Serveis Assistencials, Badalona, Barcelona, Spain.

Tamar Garzon and Manuela Lozano, Institut d’Assistència Sanitària, Girona, Spain.

Marta Cullell and Sonia Vega, Fundació Salut Empordà, Figueres, Girona, Spain.

Sílvia Alsina, Fundació Hospital de Puigcerdà, Puigcerdà, Girona, Spain.

Maria J. Maldonado-Belmonte and Susana Vazquez-Rivera, Hospital Universitario Central de la Cruz Roja San José y Santa Adela, Madrid, Spain.

Eva Baillès and Sandra Navarro, Servei Andorrà d’Atenció Sanitària (SAAS), Andorra.

## Author contributions

MA, MG, CJ, and BS designed the study. NC and NAUTILUS-Project Collaborative Group collected the data. MA performed the statistical analyses and wrote the first version of the manuscript. CJ revised the manuscript critically for important intellectual content. All authors revised the manuscript drafts and approved the final manuscript.
