# Supplementary material for: Neuropsychological impairment in post-COVID condition individuals with and without cognitive complaints
Source: Front Aging Neurosci. 2022 Oct 20;14:1029842. doi: 10.3389/fnagi.2022.1029842 (PMC9631485; doi:10.3389/fnagi.2022.1029842)
Supplement: Supplementary file 1 [file Table_1.docx]

**Supplementary table. Adjusted* means for the neuropsychological variables for PCC and HC groups**

|  | **PCC** | **HC** |  |  | |
| --- | --- | --- | --- | --- | --- |
|  | M_adj_ (SE) | M_adj_ (SE) | F | p | η_p_² |
| **MoCA** | **26.12 (0.14)** | **27.14 (0.27)** | **10.120** | **.002** | **.025** |
| Matrix reasoning | 16.25 (0.26) | 17.35 (0.47) | 3.795 | .052 | .010 |
| **RAVLT Sum** | **44.62 (0.47)** | **46.95 (0.88)** | **4.843** | **.028** | **.012** |
| RAVLT Immediate recall | 9.03 (0.16) | 9.10 (0.29) | 0.042 | .839 | .000 |
| RAVLT Delayed recall | 8.91 (0.17) | 9.24 (0.31) | 0.732 | .393 | .002 |
| RAVLT Recognition | 12.23 (0.14) | 12.57 (0.26) | 1.204 | .273 | .003 |
| ROCFT Copy | 32.94 (0.21) | 32.80 (0.40) | 0.091 | .763 | .000 |
| ROCFT Delayed recall | 19.00 (0.66) | 19.09 (0.34) | 0.012 | .913 | .000 |
| Digit Span Forward | 5.80 (0.07) | 5.85 (0.13) | 2.949 | .087 | .008 |
| Digit Span Backward | 4.43 (0.69) | 4.57 (0.13) | 0.858 | .355 | .002 |
| **Digit Symbol** | **65.46 (0.96)** | **71.29 (1.78)** | **7.448** | **.007** | **.019** |
| TMT-A | 37.13 (1.20) | 34.54 (2.23) | 0.349 | .555 | .001 |
| TMT-B | 85.80 (3.08) | 77.75 (5.69) | 1.346 | . 247 | .003 |
| Stroop Words | 94.03 (1.22) | 96.76 (2.27) | 1.108 | .316 | .003 |
| Stroop Colors | 64.88 (0.77) | 67.33 (1.43) | 2.046 | .153 | .005 |
| **Stroop Word-Colors** | **38.94 (0.58)** | **42.06 (1.08)** | **5.757** | **.017** | **.015** |
| **Phonetic fluency (PMR)** | **42.18 (0.67)** | **45.75 (1.24)** | **5.802** | **.016** | **.015** |
| **Semantic fluency (animals)** | **21.08 (0.30)** | **22.72 (0.56)** | **6.055** | **.014** | **.015** |
| BNT | 52.17 (0.29) | 52.72 (0.53) | 0.758 | .384 | .002 |
| **Eye Test** | **22.30 (0.22)** | **23.61 (0.40)** | **7.576** | **.006** | **.019** |

PCC=post-COVID condition; HC=healthy control; MoCA=Montreal Cognitive Assessment; RAVLT=Rey’s auditory verbal Learning Test; ROCFT=Rey–Osterriech Complex Figure Test; TMT=Trail making test; BNT=Boston naming test.

^*^Adjusted by years of education, estimated IQ, age, sex, Chalder Fatigue Scale, Patient Health Questionnaire -9 and General Anxiety Disorder Scale -7 scores.

η_p_² effect size is as follows: η_p_^2^=.009, small; η_p_^2^=.059, medium; η_p_^2^=.139, large.
